# Foreword to the focus issue: materials science and technology for magnetic thermal management

**DOI:** 10.1080/14686996.2025.2604891

**Published:** 2026-01-08

**Authors:** Ken-ichi Uchida, Yuya Sakuraba, Hossein Sepehri-Amin

**Affiliations:** a Research Center for Magnetic and Spintronic Materials, National Institute for Materials Science, Tsukuba, Japan; b Department of Advanced Materials Science, Graduate School of Frontier Sciences, The University of Tokyo, Kashiwa, Japan

Magnetic materials are used in various equipment and systems such as electric vehicle motors, power generators, and electronic devices, making them essential materials for human life. If omnipresent magnets could be used to directly convert heat into electricity, refrigerate other materials by applying a charge current or magnetic field, and actively control a heat flow, it would lead to innovative energy-saving and energy-creating technologies. In the Exploratory Research for Advanced Technology (ERATO) ‘UCHIDA Magnetic Thermal Management Materials’ project [1], one of the Strategic Basic Research Programs of Japan Science and Technology (JST), we are challenging the creation of future key technologies to effectively utilize the large amount of thermal energy that is being released unused, through interdisciplinary fusion studies. This Focus Issue aims to invigorate materials science contributing to thermal management technologies by simultaneously disseminating part of the achievements from this ERATO project and cutting-edge research outcomes from leading researchers worldwide in related fields.

In recent years, principles for energy conversion and control utilizing the degree of freedom of spin, the origin of magnetism, have been discovered one after another in the field of spin caloritronics, which is based on the fusion of spintronics, thermoelectrics, and thermal transport physics [2–4]. Spin caloritronics has grown rapidly since the discovery of the spin Seebeck effect [5], i.e. spin current generation by a heat current, and various thermo-spin/magneto-thermoelectric effects have been observed subsequently. Most of the research on spin caloritronics reported to date has focused on fundamental physics, and significant progress has been made in elucidating the basic mechanisms of heat-spin-charge conversion through the studies on model materials. In contrast, the development of functional materials and thermal engineering applications has been limited so far. To bring spin caloritronics into practical applications, it is essential to create innovative energy materials capable of highly efficient thermal energy conversion, control, and transfer, and to establish thermal measurement and analysis technologies for designing such materials, in addition to further investigation of heat-spin-charge conversion principles.

This Focus Issue comprises 11 original articles and 5 review articles reporting cutting-edge achievements in spin caloritronics and thermoelectrics [6–13], thermal switching/diode [14–16], magnetic refrigeration and caloric effects [17–19], and measurement technology development contributing to the advancement of these fields [20,21]. We anticipate that this Focus Issue contributes to the development of innovative materials and devices for the realization of a sustainable society.
